# [1,2-Bis(dimethyl­phosphino)ethane]carbon­yl(η^5^-cyclo­penta­dien­yl)iron(II) diphenyl­phosphinoylborate

**DOI:** 10.1107/S1600536807068651

**Published:** 2008-01-11

**Authors:** Kajin Lee, Alan J. Lough, Ian Manners

**Affiliations:** a School of Chemistry, University of Bristol, Cantock’s Close, Bristol BS8 1TS, England; b Department of Chemistry, University of Toronto, Toronto, Ontario, Canada M5S 3H6

## Abstract

In the title compound, [Fe(C_5_H_5_)(C_6_H_16_P_2_)(CO)](C_12_H_13_BOP), the Fe^II^ ion adopts a three-legged piano-stool geometry, with Fe⋯*Cg* = 1.721 (5)Å (*Cg* = the centroid defined by the C atoms of the cyclo­penta­dienyl ring). The 1,2-bis­(dimethyl­phosphino)ethane (dmpe) ligand chelates to form a five-membered C_2_P_2_Fe ring which is in a pseudo-half-chair conformation. In the crystal structure, associations of one cation and two anions are formed *via* weak inter­molecular C—H⋯O hydrogen bonds, giving rise to *R*_4_^2^(9) rings.

## Related literature

For related literature, see: Jaska *et al.* (2003 ▶, 2005 ▶); Kuckmann *et al.* (2007 ▶); Paciello *et al.* (1990 ▶). For background on graph-set theory, see: Bernstein *et al.* (1995 ▶).
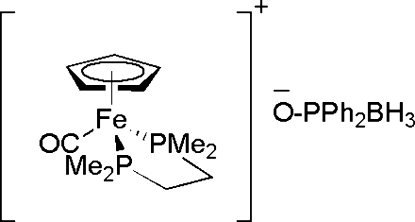


## Experimental

### 

#### Crystal data

[Fe(C_5_H_5_)(C_6_H_16_P_2_)(CO)](C_12_H_13_BOP)
*M* *_r_* = 514.08
Triclinic, 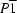

*a* = 9.0244 (5) Å
*b* = 11.4671 (4) Å
*c* = 14.0568 (7) Å
α = 67.491 (3)°
β = 81.155 (2)°
γ = 71.497 (3)°
*V* = 1273.50 (11) Å^3^
*Z* = 2
Mo *K*α radiation
μ = 0.80 mm^−1^
*T* = 150 (1) K
0.20 × 0.14 × 0.12 mm

#### Data collection

Nonius KappaCCD diffractometer
Absorption correction: multi-scan (*SORTAV*; Blessing, 1995 ▶) *T*_min_ = 0.521, *T*_max_ = 0.943
8558 measured reflections
4217 independent reflections
2935 reflections with *I* > 2σ(*I*)
*R*_int_ = 0.091

#### Refinement

*R*[*F* ^2^ > 2σ(*F* ^2^)] = 0.058
*wR*(*F* ^2^) = 0.176
*S* = 1.05
4217 reflections
296 parameters
H atoms treated by a mixture of independent and constrained refinement
Δρ_max_ = 0.81 e Å^−3^
Δρ_min_ = −0.79 e Å^−3^

### 

Data collection: *COLLECT* (Nonius, 2002 ▶); cell refinement: *DENZO-SMN* (Otwinowski & Minor, 1997 ▶); data reduction: *DENZO-SMN*; program(s) used to solve structure: *SIR92* (Altomare *et al.*, 1994 ▶); program(s) used to refine structure: *SHELXTL* (Sheldrick, 2001 ▶); molecular graphics: *PLATON* (Spek, 2003 ▶) and *SHELXTL*; software used to prepare material for publication: *SHELXTL*

## Supplementary Material

Crystal structure: contains datablocks global, I. DOI: 10.1107/S1600536807068651/hb2681sup1.cif

Structure factors: contains datablocks I. DOI: 10.1107/S1600536807068651/hb2681Isup2.hkl

Additional supplementary materials:  crystallographic information; 3D view; checkCIF report

## Figures and Tables

**Table 1:** Selected bond lengths (Å)

Fe1—C12	1.733 (5)
Fe1—P1	2.2129 (15)
Fe1—P2	2.2133 (13)

**Table 2:** Hydrogen-bond geometry (Å, °)

*D*—H⋯*A*	*D*—H	H⋯*A*	*D*⋯*A*	*D*—H⋯*A*
C2—H2*A*⋯O2^i^	1.00	2.41	3.389 (7)	167
C3—H3*A*⋯O2^ii^	1.00	2.20	3.197 (7)	172
C11—H11*B*⋯O2^ii^	0.98	2.35	3.281 (7)	159
C11—H11*C*⋯O2^i^	0.98	2.43	3.403 (7)	171

Symmetry codes: (i) 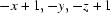; (ii) 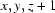.

**Fig. 1.**
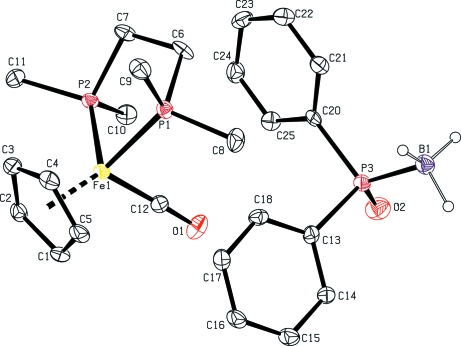
The molecular structure of (II) with displacement ellipsoids drawn at the 30% probability level. H atoms bonded to C atoms are not shown.

**Fig. 2.**
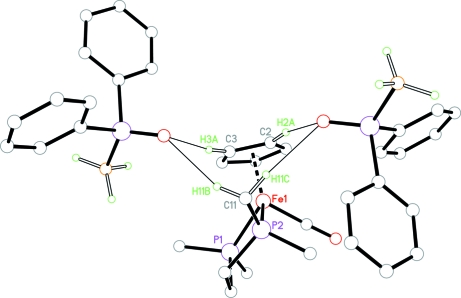
Part of the crystal structure of (II) showing hydrogen bonds as thin lines. Only the H atoms bonded to B atoms and those involved in hydrogen bonding are shown.

**Fig. 3.**
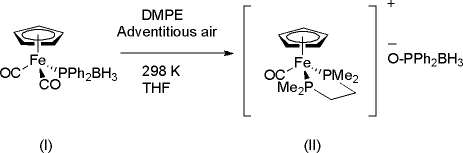
The reaction scheme.

## supplementary crystallographic information

### Comment

The mechanism for the metal catalyzed dehydrocoupling of phosphine-borane adducts has been studied by investigating the synthesis and reactivity of model complexes. The P—H bond oxidative addition of RPhPH-BH_3_ to Pt(PEt_3_)_3_ has been reported as well as the phosphine-borane ligand-exchange reaction at the Pt centre of *cis*-[PtH(PPh_2_.BH_3_) (depe)] (Jaska *et al.*, 2003). Model complexes such as *cis*-[PtH(PPhH.BH_3_)(dcype)] [dcype = 1,2-bis(dicyclohexylphosphino)ethane] have been synthesized *via* dehydrocoupling routes involving Pt—H and P—H bonds of *cis*-[PtH_2_(dcype)] and PhPH_2_.BH_3_ respectively (Jaska *et al.*, 2005). The reactivity of CpFe(CO)_2_PPh_2_.BH_3_, (I), (Kuckmann *et al.*, 2007) [see Fig. 3] was probed as a potential model complex in the study of the mechanism of the dehydrocoupling of phosphine-borane adducts: the CO ligands might dissociate to promote a reaction with phosphine-borane adducts. Before reacting (I) with phosphine-borane adducts, dmpe (1,2-bis(dimethylphosphino)ethane) was added in excess to (I) to observe the lability of the CO ligands. When adventitious air was also introduced to this reaction in THF, the title compound, (II), (Fig. 1), was formed. A similar complex, Cp*Fe(dmpe)*X* (*X* = H, CH_3_ or Cl) was reported earlier (Paciello *et al.*, 1990).

The Fe atom in (II) is bonded to a cyclopentadienyl (cp) ring with Fe···C_g_ = 1.721 (5)Å (C_g_ = the centroid of C1—C5), a carbonyl group and the bis-chelating (dimethylphosphino)ethane (dmpe) ligand (Table 1). In the crystal of (II), weak intermolecular C—H···O hydrogen bonds (Table 2) form rings with graph set assignment *R*^2^_4_(9) (Bernstein *et al.*, 1995) created in a three component cluster of two anions and one cation (Fig. 2).

### Experimental

The complex CpFe(CO)_2_PPh_2_.BH_3_ (200 mg, 0.532 mmol) was dissolved in 1.8 ml THF in a round bottom Schlenk flask. This yellow solution turned orange upon addition of dmpe (0.150 ml, 0.899 mmol). After 8 days of stirring at 293 K, orange precipitate was observed in the solution. The solution was filter cannulated into a new flask and then the volatile components of the reaction mixture were removed *in vacuo* overnight. The product was purified by chromatography with a column of celite (0.5 cm × 1.5 cm) supported on glass wool with hexanes (4 ml), Et_2_O (5 ml) and then THF (4 ml). The product in THF afforded pale yellow needles of (II) due to adventitious air.

### Refinement

All hydrogen atoms bonded to C were placed in calculated positions with C—H = 0.95–1.00 Å and refined as riding with *U*_iso_(H) = 1.2*U*_eq_(C) or 1.5*U*_eq_(C_methyl_). The H atoms bonded to B1 were refined independently with isotropic displacement parameters.

### Crystal data

**Table:**

[Fe(C_5_H_5_)(C_6_H_16_P_2_)(CO)](C_12_H_13_BOP)	*Z* = 2
*M**_r_* = 514.08	*F*_000_ = 540
Triclinic, *P*1	*D*_x_ = 1.341 Mg m^−^^3^
Hall symbol: -P 1	Mo *K*α radiation λ = 0.71073 Å
*a* = 9.0244 (5) Å	Cell parameters from 8558 reflections
*b* = 11.4671 (4) Å	θ = 2.6–25.0º
*c* = 14.0568 (7) Å	µ = 0.80 mm^−^^1^
α = 67.491 (3)º	*T* = 150 (1) K
β = 81.155 (2)º	Cut needle, pale yellow
γ = 71.497 (3)º	0.20 × 0.14 × 0.12 mm
*V* = 1273.50 (11) Å^3^	

### Data collection

**Table:**

Nonius KappaCCD diffractometer	4217 independent reflections
Radiation source: fine-focus sealed tube	2935 reflections with *I* > 2σ(*I*)
Monochromator: graphite	*R*_int_ = 0.091
Detector resolution: 9 pixels mm^-1^	θ_max_ = 25.0º
*T* = 150(1) K	θ_min_ = 2.6º
φ scans and ω scans with κ offsets	*h* = −10→10
Absorption correction: multi-scan(SORTAV; Blessing, 1995)	*k* = −12→13
*T*_min_ = 0.521, *T*_max_ = 0.943	*l* = −16→16
8558 measured reflections	

### Refinement

**Table:**

Refinement on *F*^2^	Secondary atom site location: difference Fourier map
Least-squares matrix: full	Hydrogen site location: inferred from neighbouring sites
*R*[*F*^2^ > 2σ(*F*^2^)] = 0.058	H atoms treated by a mixture of independent and constrained refinement
*wR*(*F*^2^) = 0.176	*w* = 1/[σ^2^(*F*_o_^2^) + (0.0844*P*)^2^ + 1.3773*P*] where *P* = (*F*_o_^2^ + 2*F*_c_^2^)/3
*S* = 1.05	(Δ/σ)_max_ = 0.002
4217 reflections	Δρ_max_ = 0.81 e Å^−^^3^
296 parameters	Δρ_min_ = −0.79 e Å^−^^3^
Primary atom site location: structure-invariant direct methods	Extinction correction: none

### Special details

**Table:**

Geometry. All e.s.d.'s (except the e.s.d. in the dihedral angle between two l.s. planes) are estimated using the full covariance matrix. The cell e.s.d.'s are taken into account individually in the estimation of e.s.d.'s in distances, angles and torsion angles; correlations between e.s.d.'s in cell parameters are only used when they are defined by crystal symmetry. An approximate (isotropic) treatment of cell e.s.d.'s is used for estimating e.s.d.'s involving l.s. planes.
Refinement. Refinement of *F*^2^ against ALL reflections. The weighted *R*-factor *wR* and goodness of fit *S* are based on *F*^2^, conventional *R*-factors *R* are based on *F*, with *F* set to zero for negative *F*^2^. The threshold expression of *F*^2^ > σ(*F*^2^) is used only for calculating *R*-factors(gt) *etc*. and is not relevant to the choice of reflections for refinement. *R*-factors based on *F*^2^ are statistically about twice as large as those based on *F*, and *R*- factors based on ALL data will be even larger.

### Fractional atomic coordinates and isotropic or equivalent isotropic displacement parameters (Å^2^)

**Table:**

	*x*	*y*	*z*	*U*_iso_*/*U*_eq_	
Fe1	0.22249 (8)	0.31967 (6)	0.79005 (5)	0.0227 (2)	
P1	−0.03606 (15)	0.38455 (12)	0.78938 (10)	0.0256 (3)	
P2	0.20337 (15)	0.11946 (11)	0.82546 (10)	0.0231 (3)	
O1	0.2462 (5)	0.3727 (4)	0.5707 (3)	0.0444 (10)	
C1	0.4180 (6)	0.3862 (5)	0.7772 (4)	0.0339 (13)	
H1A	0.4862	0.4108	0.7139	0.041*	
C2	0.4403 (6)	0.2587 (5)	0.8551 (4)	0.0294 (12)	
H2A	0.5270	0.1779	0.8568	0.035*	
C3	0.3203 (6)	0.2692 (5)	0.9321 (4)	0.0319 (13)	
H3A	0.3063	0.1965	0.9974	0.038*	
C4	0.2271 (6)	0.4001 (5)	0.9012 (4)	0.0305 (12)	
H4A	0.1336	0.4357	0.9409	0.037*	
C5	0.2834 (6)	0.4729 (5)	0.8063 (4)	0.0326 (13)	
H5A	0.2400	0.5691	0.7674	0.039*	
C6	−0.1017 (6)	0.2512 (5)	0.7849 (4)	0.0328 (12)	
H6A	−0.2134	0.2631	0.8071	0.039*	
H6B	−0.0893	0.2506	0.7138	0.039*	
C7	−0.0015 (6)	0.1229 (5)	0.8573 (4)	0.0301 (12)	
H7A	−0.0212	0.0471	0.8492	0.036*	
H7B	−0.0275	0.1172	0.9297	0.036*	
C8	−0.1254 (7)	0.5297 (5)	0.6827 (4)	0.0397 (14)	
H8A	−0.2394	0.5477	0.6902	0.060*	
H8B	−0.0891	0.5149	0.6178	0.060*	
H8C	−0.0961	0.6052	0.6824	0.060*	
C9	−0.1446 (6)	0.4183 (6)	0.9003 (4)	0.0372 (13)	
H9A	−0.2544	0.4233	0.8977	0.056*	
H9B	−0.1377	0.5022	0.8996	0.056*	
H9C	−0.1003	0.3476	0.9636	0.056*	
C10	0.2542 (6)	0.0595 (5)	0.7200 (4)	0.0337 (13)	
H10A	0.2284	−0.0237	0.7398	0.051*	
H10B	0.3665	0.0453	0.7031	0.051*	
H10C	0.1952	0.1244	0.6596	0.051*	
C11	0.3108 (6)	−0.0181 (5)	0.9308 (4)	0.0318 (12)	
H11A	0.2859	−0.0984	0.9380	0.048*	
H11B	0.2812	−0.0001	0.9950	0.048*	
H11C	0.4233	−0.0300	0.9163	0.048*	
C12	0.2342 (6)	0.3517 (4)	0.6586 (4)	0.0275 (12)	
P3	0.21995 (16)	0.08074 (12)	0.22685 (10)	0.0265 (3)	
O2	0.3092 (5)	0.0325 (4)	0.1455 (3)	0.0482 (11)	
C13	0.3424 (6)	0.1487 (4)	0.2728 (4)	0.0246 (11)	
C14	0.4211 (6)	0.2322 (5)	0.1986 (4)	0.0287 (12)	
H14A	0.4080	0.2534	0.1276	0.034*	
C15	0.5184 (6)	0.2844 (5)	0.2277 (4)	0.0346 (13)	
H15A	0.5714	0.3412	0.1765	0.042*	
C16	0.5383 (6)	0.2541 (5)	0.3308 (4)	0.0332 (12)	
H16A	0.6064	0.2886	0.3505	0.040*	
C17	0.4598 (6)	0.1742 (5)	0.4046 (4)	0.0328 (13)	
H17A	0.4721	0.1550	0.4754	0.039*	
C18	0.3619 (6)	0.1208 (5)	0.3767 (4)	0.0283 (12)	
H18A	0.3082	0.0653	0.4285	0.034*	
C20	0.2031 (6)	−0.0630 (4)	0.3411 (4)	0.0249 (11)	
C21	0.0648 (6)	−0.0653 (5)	0.4012 (4)	0.0303 (12)	
H21A	−0.0229	0.0107	0.3858	0.036*	
C22	0.0560 (7)	−0.1787 (5)	0.4833 (4)	0.0355 (13)	
H22A	−0.0384	−0.1800	0.5238	0.043*	
C23	0.1819 (7)	−0.2897 (5)	0.5070 (4)	0.0348 (13)	
H23A	0.1741	−0.3672	0.5631	0.042*	
C24	0.3195 (6)	−0.2878 (5)	0.4489 (4)	0.0351 (13)	
H24A	0.4072	−0.3637	0.4659	0.042*	
C25	0.3308 (6)	−0.1761 (5)	0.3662 (4)	0.0310 (12)	
H25A	0.4257	−0.1761	0.3262	0.037*	
B1	0.0223 (8)	0.2119 (6)	0.1944 (5)	0.0338 (14)	
H1	0.045 (6)	0.298 (5)	0.132 (4)	0.030 (13)*	
H2	−0.037 (7)	0.166 (6)	0.160 (5)	0.062 (18)*	
H3	−0.020 (6)	0.244 (5)	0.265 (4)	0.043 (15)*	

### Atomic displacement parameters (Å^2^)

**Table:**

	*U*^11^	*U*^22^	*U*^33^	*U*^12^	*U*^13^	*U*^23^
Fe1	0.0241 (4)	0.0196 (4)	0.0260 (4)	−0.0091 (3)	−0.0030 (3)	−0.0068 (3)
P1	0.0240 (7)	0.0230 (7)	0.0301 (7)	−0.0048 (5)	−0.0049 (6)	−0.0097 (6)
P2	0.0233 (7)	0.0193 (7)	0.0280 (7)	−0.0083 (5)	−0.0021 (5)	−0.0077 (5)
O1	0.061 (3)	0.040 (2)	0.027 (2)	−0.012 (2)	−0.0033 (19)	−0.0081 (17)
C1	0.033 (3)	0.037 (3)	0.037 (3)	−0.023 (3)	−0.003 (2)	−0.007 (2)
C2	0.030 (3)	0.024 (3)	0.037 (3)	−0.008 (2)	−0.011 (2)	−0.009 (2)
C3	0.045 (3)	0.032 (3)	0.029 (3)	−0.024 (3)	−0.007 (3)	−0.008 (2)
C4	0.034 (3)	0.036 (3)	0.035 (3)	−0.015 (2)	0.001 (2)	−0.024 (2)
C5	0.034 (3)	0.026 (3)	0.046 (3)	−0.015 (2)	−0.007 (3)	−0.014 (2)
C6	0.018 (3)	0.039 (3)	0.052 (3)	−0.012 (2)	−0.002 (2)	−0.025 (3)
C7	0.025 (3)	0.028 (3)	0.045 (3)	−0.013 (2)	0.001 (2)	−0.017 (2)
C8	0.034 (3)	0.033 (3)	0.038 (3)	0.004 (2)	−0.011 (3)	−0.006 (2)
C9	0.029 (3)	0.050 (3)	0.038 (3)	−0.011 (3)	0.005 (2)	−0.024 (3)
C10	0.040 (3)	0.024 (3)	0.039 (3)	−0.010 (2)	−0.003 (3)	−0.013 (2)
C11	0.037 (3)	0.021 (3)	0.034 (3)	−0.009 (2)	−0.005 (2)	−0.004 (2)
C12	0.031 (3)	0.017 (3)	0.034 (3)	−0.008 (2)	−0.007 (2)	−0.005 (2)
P3	0.0302 (8)	0.0211 (7)	0.0302 (7)	−0.0117 (6)	−0.0063 (6)	−0.0059 (5)
O2	0.058 (3)	0.043 (2)	0.048 (3)	−0.021 (2)	0.001 (2)	−0.0176 (19)
C13	0.027 (3)	0.017 (3)	0.028 (3)	−0.003 (2)	−0.006 (2)	−0.007 (2)
C14	0.032 (3)	0.025 (3)	0.029 (3)	−0.012 (2)	−0.004 (2)	−0.006 (2)
C15	0.036 (3)	0.030 (3)	0.042 (3)	−0.017 (2)	−0.002 (3)	−0.011 (2)
C16	0.034 (3)	0.030 (3)	0.044 (3)	−0.016 (2)	−0.007 (3)	−0.014 (2)
C17	0.037 (3)	0.027 (3)	0.035 (3)	−0.007 (2)	−0.012 (2)	−0.009 (2)
C18	0.029 (3)	0.021 (3)	0.032 (3)	−0.009 (2)	0.002 (2)	−0.007 (2)
C20	0.028 (3)	0.027 (3)	0.028 (3)	−0.015 (2)	−0.008 (2)	−0.011 (2)
C21	0.025 (3)	0.029 (3)	0.038 (3)	−0.008 (2)	−0.003 (2)	−0.012 (2)
C22	0.032 (3)	0.037 (3)	0.038 (3)	−0.015 (3)	0.004 (2)	−0.012 (2)
C23	0.048 (4)	0.030 (3)	0.027 (3)	−0.021 (3)	0.002 (3)	−0.005 (2)
C24	0.036 (3)	0.028 (3)	0.038 (3)	−0.005 (2)	−0.011 (3)	−0.008 (2)
C25	0.031 (3)	0.029 (3)	0.033 (3)	−0.008 (2)	0.000 (2)	−0.012 (2)
B1	0.034 (4)	0.029 (3)	0.039 (4)	−0.010 (3)	−0.007 (3)	−0.010 (3)

### Geometric parameters (Å, °)

**Table:**

Fe1—C12	1.733 (5)	C10—H10A	0.9800
Fe1—P1	2.2129 (15)	C10—H10B	0.9800
Fe1—P2	2.2133 (13)	C10—H10C	0.9800
Fe1—C2	2.093 (5)	C11—H11A	0.9800
Fe1—C1	2.093 (5)	C11—H11B	0.9800
Fe1—C5	2.097 (5)	C11—H11C	0.9800
Fe1—C3	2.105 (5)	P3—O2	1.477 (4)
Fe1—C4	2.107 (5)	P3—C20	1.837 (5)
P1—C8	1.811 (5)	P3—C13	1.844 (5)
P1—C9	1.815 (5)	P3—B1	1.917 (6)
P1—C6	1.831 (5)	C13—C18	1.396 (7)
P2—C10	1.804 (5)	C13—C14	1.396 (7)
P2—C11	1.818 (5)	C14—C15	1.388 (7)
P2—C7	1.826 (5)	C14—H14A	0.9500
O1—C12	1.160 (6)	C15—C16	1.382 (7)
C1—C5	1.422 (7)	C15—H15A	0.9500
C1—C2	1.426 (7)	C16—C17	1.369 (7)
C1—H1A	1.0000	C16—H16A	0.9500
C2—C3	1.418 (7)	C17—C18	1.394 (7)
C2—H2A	1.0000	C17—H17A	0.9500
C3—C4	1.401 (7)	C18—H18A	0.9500
C3—H3A	1.0000	C20—C21	1.397 (7)
C4—C5	1.394 (7)	C20—C25	1.401 (7)
C4—H4A	1.0000	C21—C22	1.385 (7)
C5—H5A	1.0000	C21—H21A	0.9500
C6—C7	1.521 (7)	C22—C23	1.377 (8)
C6—H6A	0.9900	C22—H22A	0.9500
C6—H6B	0.9900	C23—C24	1.379 (7)
C7—H7A	0.9900	C23—H23A	0.9500
C7—H7B	0.9900	C24—C25	1.381 (7)
C8—H8A	0.9800	C24—H24A	0.9500
C8—H8B	0.9800	C25—H25A	0.9500
C8—H8C	0.9800	B1—H1	1.09 (5)
C9—H9A	0.9800	B1—H2	1.12 (6)
C9—H9B	0.9800	B1—H3	1.15 (5)
C9—H9C	0.9800		
			
C12—Fe1—C2	113.6 (2)	P2—C7—H7A	110.1
C12—Fe1—C1	90.6 (2)	C6—C7—H7B	110.1
C2—Fe1—C1	39.84 (19)	P2—C7—H7B	110.1
C12—Fe1—C5	105.3 (2)	H7A—C7—H7B	108.4
C2—Fe1—C5	66.7 (2)	P1—C8—H8A	109.5
C1—Fe1—C5	39.7 (2)	P1—C8—H8B	109.5
C12—Fe1—C3	153.0 (2)	H8A—C8—H8B	109.5
C2—Fe1—C3	39.5 (2)	P1—C8—H8C	109.5
C1—Fe1—C3	66.1 (2)	H8A—C8—H8C	109.5
C5—Fe1—C3	65.9 (2)	H8B—C8—H8C	109.5
C12—Fe1—C4	143.2 (2)	P1—C9—H9A	109.5
C2—Fe1—C4	65.7 (2)	P1—C9—H9B	109.5
C1—Fe1—C4	65.4 (2)	H9A—C9—H9B	109.5
C5—Fe1—C4	38.7 (2)	P1—C9—H9C	109.5
C3—Fe1—C4	38.9 (2)	H9A—C9—H9C	109.5
C12—Fe1—P1	91.01 (17)	H9B—C9—H9C	109.5
C2—Fe1—P1	155.10 (15)	P2—C10—H10A	109.5
C1—Fe1—P1	142.78 (15)	P2—C10—H10B	109.5
C5—Fe1—P1	104.63 (15)	H10A—C10—H10B	109.5
C3—Fe1—P1	115.69 (16)	P2—C10—H10C	109.5
C4—Fe1—P1	92.67 (15)	H10A—C10—H10C	109.5
C12—Fe1—P2	91.92 (15)	H10B—C10—H10C	109.5
C2—Fe1—P2	95.99 (14)	P2—C11—H11A	109.5
C1—Fe1—P2	130.86 (15)	P2—C11—H11B	109.5
C5—Fe1—P2	159.26 (15)	H11A—C11—H11B	109.5
C3—Fe1—P2	93.58 (14)	P2—C11—H11C	109.5
C4—Fe1—P2	124.82 (15)	H11A—C11—H11C	109.5
P1—Fe1—P2	86.24 (5)	H11B—C11—H11C	109.5
C8—P1—C9	102.6 (3)	O1—C12—Fe1	178.2 (5)
C8—P1—C6	106.0 (3)	O2—P3—C20	107.9 (2)
C9—P1—C6	103.3 (3)	O2—P3—C13	108.6 (2)
C8—P1—Fe1	117.0 (2)	C20—P3—C13	101.9 (2)
C9—P1—Fe1	118.65 (19)	O2—P3—B1	118.6 (3)
C6—P1—Fe1	107.95 (17)	C20—P3—B1	111.6 (3)
C10—P2—C11	102.7 (2)	C13—P3—B1	106.9 (2)
C10—P2—C7	104.4 (2)	C18—C13—C14	118.5 (4)
C11—P2—C7	104.9 (2)	C18—C13—P3	124.1 (4)
C10—P2—Fe1	115.38 (18)	C14—C13—P3	117.5 (4)
C11—P2—Fe1	119.88 (17)	C15—C14—C13	120.6 (5)
C7—P2—Fe1	108.14 (16)	C15—C14—H14A	119.7
C5—C1—C2	108.0 (5)	C13—C14—H14A	119.7
C5—C1—Fe1	70.3 (3)	C16—C15—C14	120.2 (5)
C2—C1—Fe1	70.1 (3)	C16—C15—H15A	119.9
C5—C1—H1A	126.0	C14—C15—H15A	119.9
C2—C1—H1A	126.0	C17—C16—C15	120.0 (5)
Fe1—C1—H1A	126.0	C17—C16—H16A	120.0
C3—C2—C1	107.2 (5)	C15—C16—H16A	120.0
C3—C2—Fe1	70.7 (3)	C16—C17—C18	120.5 (5)
C1—C2—Fe1	70.1 (3)	C16—C17—H17A	119.7
C3—C2—H2A	126.4	C18—C17—H17A	119.7
C1—C2—H2A	126.4	C17—C18—C13	120.2 (5)
Fe1—C2—H2A	126.4	C17—C18—H18A	119.9
C4—C3—C2	107.8 (4)	C13—C18—H18A	119.9
C4—C3—Fe1	70.7 (3)	C21—C20—C25	118.8 (4)
C2—C3—Fe1	69.8 (3)	C21—C20—P3	122.2 (4)
C4—C3—H3A	126.1	C25—C20—P3	118.9 (4)
C2—C3—H3A	126.1	C22—C21—C20	119.7 (5)
Fe1—C3—H3A	126.1	C22—C21—H21A	120.1
C5—C4—C3	109.6 (5)	C20—C21—H21A	120.1
C5—C4—Fe1	70.3 (3)	C23—C22—C21	121.0 (5)
C3—C4—Fe1	70.5 (3)	C23—C22—H22A	119.5
C5—C4—H4A	125.2	C21—C22—H22A	119.5
C3—C4—H4A	125.2	C22—C23—C24	119.6 (5)
Fe1—C4—H4A	125.2	C22—C23—H23A	120.2
C4—C5—C1	107.3 (5)	C24—C23—H23A	120.2
C4—C5—Fe1	71.0 (3)	C23—C24—C25	120.5 (5)
C1—C5—Fe1	70.0 (3)	C23—C24—H24A	119.8
C4—C5—H5A	126.3	C25—C24—H24A	119.8
C1—C5—H5A	126.3	C24—C25—C20	120.4 (5)
Fe1—C5—H5A	126.3	C24—C25—H25A	119.8
C7—C6—P1	107.3 (3)	C20—C25—H25A	119.8
C7—C6—H6A	110.3	P3—B1—H1	107 (3)
P1—C6—H6A	110.3	P3—B1—H2	101 (3)
C7—C6—H6B	110.3	H1—B1—H2	107 (4)
P1—C6—H6B	110.3	P3—B1—H3	107 (3)
H6A—C6—H6B	108.5	H1—B1—H3	106 (4)
C6—C7—P2	108.1 (3)	H2—B1—H3	127 (4)
C6—C7—H7A	110.1		

### Hydrogen-bond geometry (Å, °)

**Table:**

*D*—H···*A*	*D*—H	H···*A*	*D*···*A*	*D*—H···*A*
C2—H2A···O2^i^	1.00	2.41	3.389 (7)	167
C3—H3A···O2^ii^	1.00	2.20	3.197 (7)	172
C11—H11B···O2^ii^	0.98	2.35	3.281 (7)	159
C11—H11C···O2^i^	0.98	2.43	3.403 (7)	171

Symmetry codes: (i) −*x*+1, −*y*, −*z*+1; (ii) *x*, *y*, *z*+1.
